# Invasive Plant Species Establishment and Range Dynamics in Sri Lanka under Climate Change

**DOI:** 10.3390/e21060571

**Published:** 2019-06-05

**Authors:** Champika S. Kariyawasam, Lalit Kumar, Sujith S. Ratnayake

**Affiliations:** 1 Ecosystem Management, School of Environmental and Rural Science, University of New England, Armidale, NSW 2351, Australia; 2 Climate Change Secretariat, Ministry of Mahaweli Development and Environment, Battaramulla 10120, Sri Lanka

**Keywords:** biological invasions, climate suitability, conservation planning, MaxEnt, niche modeling, risk assessment

## Abstract

Plant invasion has been widely recognized as an agent of global change that has the potential to have severe impacts under climate change. The challenges posed by invasive alien plant species (IAPS) on biodiversity and ecosystem stability is growing and not adequately studied, especially in developing countries. Defining climate suitability for multiple invasive plants establishment is important for early and strategic interventions to control and manage plant invasions. We modeled priority IAPS in Sri Lanka to identify the areas of greatest climatic suitability for their establishment and observed how these areas could be altered under projected climate change. We used Maximum Entropy method to model 14 nationally significant IAPS under representative concentration pathways 4.5 and 8.5 for 2050 and 2070. The combined climate suitability map produced by summing up climatic suitability of 14 IAPS was further classified into five classes in ArcMap as very high, high, moderate, low, and very low. South and west parts of Sri Lanka are projected to have potentially higher climatic suitability for a larger number of IAPS. We observed suitable area changes (gains and losses) in all five classes of which two were significant enough to make an overall negative impact i.e., (i) contraction of the very low class and (ii) expansion of the moderate class. Both these changes trigger the potential risk from IAPS in Sri Lanka in the future.

## 1. Introduction

Species invasion has been recognized as an agent of global change that has the potential to make large impacts on biodiversity and associated ecological processes [1]. It is considered as a serious threat to biodiversity, second only to habitat fragmentation and degradation [2]. Invasive species can have interactions with other drivers of global change and make complex and combined effects [3]. The threat will be intensified in the future allowing more than 17% of lands vulnerable to species invasion globally [4]. The species diversity on Earth is diminishing due to the introduction of invasive species to new ranges, which is increasing and already beyond the accepted level [5]. This is a common problem everywhere in the world, continents, oceanic islands, temperate and tropical regions [6]. The problem is severe in oceanic islands where the species and ecosystems are unique and vulnerable to external impacts [7,8]. Moreover, species invasions can have negative impacts across several sectors other than biodiversity, such as agriculture, tourism, forestry, fishery, human health, water and irrigation. The ultimate impacts can be interconnected [9], resulting in an utmost threat to the economy, social and environmental stability and sustainability of the planet [10].

The challenges posed by invasive alien plant species (IAPS) on biodiversity, ecological processes and ecosystem services, particularly in island countries, is increasing and well recognized [11]. This issue continues to be a challenge and increasingly important regardless of rigorous control and management efforts. Species invasion has been facilitated by the rapid growth of trade and transportation between countries, especially after the industrial revolution [12]. The natural barriers (i.e., mountains, rivers) that provided isolation required for unique species to evolve is no longer effective [7]. Understanding the current and potential distribution patterns is fundamental for managing IAPS [13,14,15]. Species distribution models (SDMs), which are based on ecological assumptions and theories, investigate how species shift their ecological niche spatially and temporally under climate change [16,17,18,19]. Hutchinson defined the niche as n-dimensional hyper-volume within which a species can survive and reproduce; in the absence of biotic interactions this volume is equal to the species’ fundamental niche [16]. However, under given circumstances, a species will usually only occupy a certain part of the fundamental niche, which is called the realized niche [20]. Therefore, theoretically, SDMs estimate a species’ potential distribution rather than the actual distribution. When the species niche is projected to a geographical space, it yields a predictive map of species’ presence [21,22]. But there is a clear uncertainty associated with the changing climate which could be due to several reasons, such as the assumptions underlying SDMs or uncertainty associated with general circulation models (GCMs), modeling algorithms, spatial scales and the data used [17]. 

Warming of the earth’s surface has increased significantly and it will continue to warm. The global mean surface temperature will increase significantly by the end of this century (2081–2100) compared to the current period (1986–2005) under different greenhouse gas emission scenarios [23]. Accordingly, this temperature increase can range from a minimum of 0.3 °C (under RCP2.6) to a maximum of 4.8 °C (under RCP8.5). Temperature and precipitation have been reported to be the key factors determining the vegetation dynamics [24]. Climatic variables which are derivatives of meteorological data (i.e., temperature and precipitation) should result in a clear relationship with the spatial distribution of plants [25]. As the key determinant of species distribution, climate controls the geographical distribution of plants predominantly over the other non-climatic factors [26]. Climate models based on the bioclimate envelope rely on climatic variables and do not consider the non-climatic variables (i.e., soil type) [27]. However, the contribution of ecologically meaningful non-climatic variables (i.e., soil, elevation, and slope) and biotic factors (i.e., competition, predation, and pollination) has been frequently discussed in predictive modeling literature [28,29]. 

Scientists believe that global warming and associated observed changes (i.e., sea level rise, floods, saltwater intrusion) are undeniable and can make a significant impact on life on earth, the sustainability of biodiversity and ecosystem processes [10]. According to IPCC [23] medium to high emission scenarios can cause sudden, high impact, irreversible damages to the ecosystem composition, structure and function. Global climate change influences the spatial distribution of many plants in the geographic space [30,31]. When climate change happens, species that are vulnerable and unable to shift their ranges for survival can become extinct from the habitat [23]. Stress conditions created by climate change can open more favorable habitats for invasive plants [10] as they can survive in a broader range of climate conditions due to their wide environmental tolerance [32]. 

The species response to climate has been successfully used for the development of a number of species distribution models (SDMs) to assess the relative suitability of habitats for species [33]. SDMs predict species fundamental ecological niche or the area occupied by the species in the absence of interactions between living organisms [34]. The geographic distribution of species is constrained by several factors such as biotic interactions, dispersal ability, evolutionary history, geographical obstacles or human influences [21,35]. Thus, the species cannot occupy the entire fundamental niche though the climate is suitable and therefore the fundamental niche is generally larger than the realized niche that the species’ actually occupies [36]. This explains that the prediction can be mostly an over-prediction of the area than the species can establish and spread. SDMs can be projected under changing climate to different geographical areas or time periods to forecast the potential area of spread or assess invasion risk of a species [21]. Relevant to native species, SDMs can be used to assess potentially vulnerable species and sites under climate change [37]. This has made SDMs more important in applied ecology to understand species response in the current and future climate [38] to develop effective and appropriate strategies for conservation and management. A large number of studies use SDMs to predict potential areas of distribution. Literature shows the importance of updated climate change projections using multiple scenarios as many distribution studies are based on older versions of climate change projections and a significant number of them cover only one climate change scenario [39].

We examined the climatic suitability for multiple IAPS establishment and potential invasion dynamics in tropical island settings under climate change using Sri Lanka as an example. Like many tropical islands, Sri Lanka is rich in biodiversity with high conservation values which is at risk by plant invaders. The aims of present study were to (i) identify the potential climate suitability for multiple IAPS establishment under the current climate; (ii) predict potential changes by 2050 and 2070 under two climate change scenarios; (iii) classify climate suitability of the country and examine potential invasion dynamics under climate change. We modeled 14 IAPS which are at different stages of invasion using MaxEnt niche model. More specifically we identified the areas susceptible to multiple IAPS establishment and how will this susceptibility change under the projected climate change. Our study is significant due to two important reasons. Firstly, this study focused on the multi-species projection which had been the focus of a limited number of modeling studies. Secondly, hitherto no comprehensive study has been done to forecast the potential distribution of IAPS under projected climate change in tropical island countries in the region with similar bioclimatic conditions. Defining climatic suitability for multiple IAPS invasion is important for their successful control and management. 

## 2. Materials and Methods

### 2.1. Study Area

Sri Lanka is an island country located in the Indian Ocean, between 5°54′ and 9°52′ north latitude and 79°39′ and 81°53′ east longitude, with an approximate area of 65,610 km^2^. Topographically, the country is divided into three peneplains [40]. The mountainous region located in the south-central part of Sri Lanka rising above 2500 m is surrounded by broad lowland plains [41]. The climate of Sri Lanka is generally tropical and warm; however, a wide variation can be observed across the country due to the differences in rainfall and elevation [41]. Sri Lanka is endowed with diverse vegetation types mainly due to variations in topography, climate, soil and also coastal influence [42]. The unique and diverse ecosystems have provided habitats for rich species diversity and genetic variability among species [41]. Out of the total land area of the country, nearly 35% is reserved for conservation purposes [40] and around 40% is utilized for agriculture [43]. 

### 2.2. Modeling Method

We used MaxEnt or the Maximum entropy species distribution modeling [21] which is one of the most popular [38] and globally accepted correlative modeling technique currently in use for predicting the potential distribution of species [44,45]. MaxEnt predicts potential areas of suitability of species better than many other well-established species distribution methods using presence-only occurrence data, environmental variables and background records across the defined geographic area [46]. MaxEnt is underpinned on the maximum entropy (i.e., meaning most spread out or closest to uniform distribution) principle [21]. While running the model, MaxEnt estimates the probability distribution of maximum entropy of environmental variables across the entire study area which is represented by a number of grid cells [44,47] and the species niche is defined relevant to these variables or features. MaxEnt has been used in many applications in conservation science today, particularly on invasive species [48]. For instance, MaxEnt has been widely used to model the ecological niches of invasive species to predict areas where an invasive species could potentially occur in the future [49,50]. The focus of these studies is to examine the potential distribution of selected species to classify areas for future strategic management. Other popular applications of MaxEnt are reserve planning, identify critical habitats for threatened and endangered species, ecological risk assessment, species translocation, climate change impacts [21,30,51,52].

### 2.3. Species Occurrence Data

**Figure 1:**
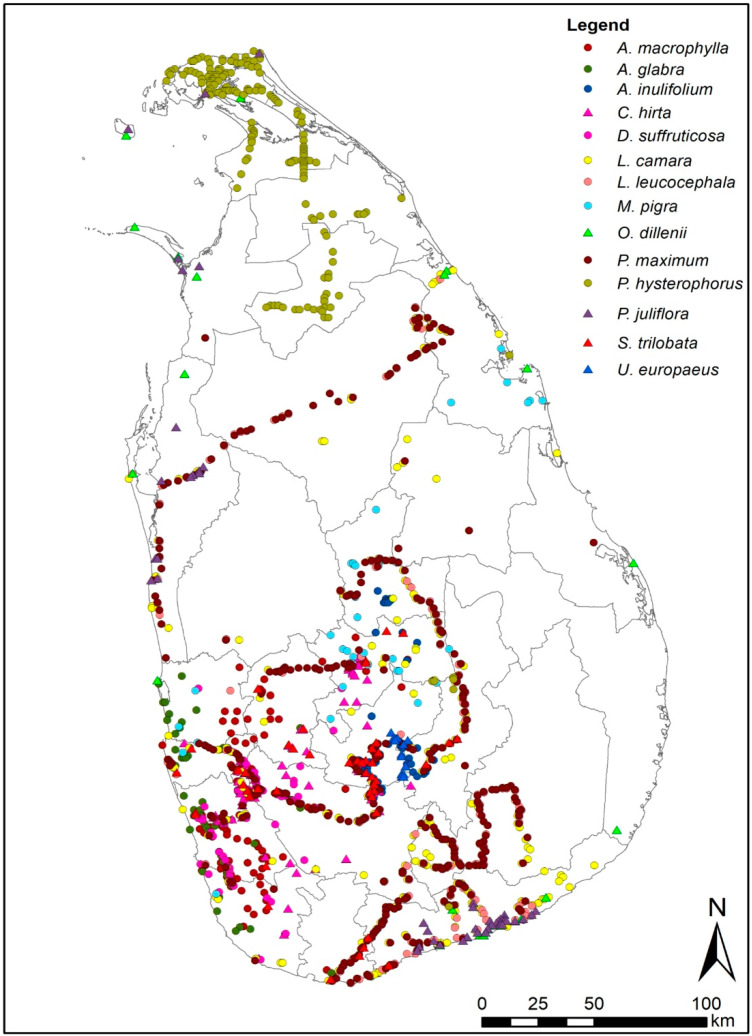
Distribution of occurrence records used for MaxEnt modeling of 14 priority IAPS in Sri Lanka. The above species records were extracted from several published literature, online sources and expert communications [53,58,59,60,61,62].

In 2015 the Government of Sri Lanka identified 20 IAPS of national significance based on the criteria developed through a wider participatory and consultative process [53]. This list includes 16 terrestrial IAPS. The present study modeled only 14 IAPS (Table 1) out of the 16 terrestrial IAPS included in the national list of invasive plants of Sri Lanka (2 species were excluded due to the inadequacy of data on occurrences). We did not consider aquatic IAPS as modeling aquatic plants is relatively difficult and not common, perhaps due to the complexity of the interaction of environmental factors [54] that influence growth and spread of aquatic plants. Geo-referenced occurrence records were obtained from either published or grey literature for the 14 evaluated species (Figure 1). Most of the records in databases do not contain the sampling year(s) of occurrence data collected; however, these records approximately represent the period 1960–2015. MaxEnt models are underpinned on the assumption that all locations (more importantly in the environmental space) are uniformly sampled [55]. Therefore, all occurrences were filtered at 30 s (0.0083°) grid cell to remove the spatial sampling bias and thus to improve model performance [56,57]. Spatial filtering reduced the total occurrences of 14 species to 1460, resulting in one occurrence for each grid cell in the areas where occurrences are spatially distributed.

### 2.4. Environmental Predictors

We decided to undertake modeling exclusively on climate data due to the following reasons (i) climate provides good prediction over the distribution of invasive species and most other variables (i.e., elevation) are proxy variables for climate; (ii) studies have successfully verified a similarity between the climatic conditions of the ecological habitat and the pattern of spread of invasive species [33,63]; and iii) categorical variables are less compatible with continuous variables in MaxEnt model [64]. Therefore, we selected Worldclim (version 1.4), 19 bioclimatic variables at a resolution of 30 arc-seconds (~1 km^2^) for current (representative of 1960–1990) and future [65] (http://www.worldclim.org). These variables have the capacity to determine the spatial limits of climatic tolerance of the species and hence widely used for species distribution modeling studies. 

For future projections, we used an improved fifth version of the atmosphere-ocean general circulation model (GCM), Model for Interdisciplinary Research on Climate (MIROC). These data were downloaded from the Worldclim website. MIROC5, which was used for the Intergovernmental Panel on Climate Change (IPCC) Fifth Assessment Report (AR5), is an important tool with significantly improved climatological features for better performance of climate change simulations [30,66] particularly in the South Asian region [49]. We selected RCPs (Representative Concentration Pathways) 4.5 and RCP 8.5 greenhouse gas concentration trajectories for 2050 and 2070. RCP 4.5 (emissions peaks around 2040 and then decline) provides the intermediate scenario of greenhouse gas emissions, atmospheric concentrations, air pollutant emissions and land use changes in the future while RCP 8.5 (emissions rapidly rise until 2100) provides a very high scenario [23].

Removing highly correlated variables are recommended in predictive modeling [55,67] as variables can be correlated and the combined effect may change the model’s performance, resulting in inaccurate and misleading predictions [68]. Pearson Correlation Coefficients (r) among 19 bioclim variables were calculated using ‘removeCollinearity’ function of the Package ‘virtualspecies’ (version 1.4-4) in R [69]. Variables with a correlation value of r ≥0.7 were excluded (Supplementary material S1). This threshold level is an acceptable measure to minimize multicollinearity of the fitted models [68]. This resulted in variables into seven groups of intercorrelated variables. A subset of seven non-correlated environmental variables was selected for the model run (Table 2). While selecting variables, special attention was paid to select variables that were highly responsive for a plausible forecast of species relationship to climate change and widely used in invasive species modeling literature [70,71,72]. 

### 2.5. MaxEnt Settings

We used MaxEnt version 3.4.1 [73] (http://biodiversityinformatics.amnh.org/open_source/maxent/) for the current modeling study. Selection of MaxEnt approach was based on several well-known reasons (i) MaxEnt is relatively more robust over the other methods for small sample sizes [51,74,75,76]; (ii) MaxEnt is resistant to spatial errors in occurrence data up to a certain extent [51] and performs well with occurrences that show sampling bias [77]; (iii) MaxEnt is more popular for predicting potential ranges of invasive plants [21,55] and previously used for defining invasive plant hotspots [63,71]; (vi) generally, simple models are more applicable for modeling IAPS and MaxEnt settings can be changed to make the model smoother (i.e., feature types) [78] and (vii) MaxEnt generates a continuous output, facilitating distinct binary predictions which is imperative in IAPS modeling [21,75]. Logistic output, which is depicted by a linear scale of values ranging from 0 to 1, was selected for easy interpretation of results [35]. Background records (10,000) were used across the country as several species are used and the distribution of them in the landscape is not correctly known [55]. Cross-validation was employed with 10 replicates as we had relatively smaller sample sizes for some species. This enables MaxEnt to use all data efficiently in model fitting and to receive a more realistic average output. Concurrently, it helps to evaluate the model uncertainty [55]. However, we understand that lack of validation of the independent dataset can cause biased predictions generated by unknown overfitting [79,80]. We also used 1000 maximum iterations in our models, enabling the algorithm to get close to convergence for a realistic prediction [21]. Linear, quadratic and hinge features were used since sample sizes were relatively low. All other default values of user-specified parameters of MaxEnt relating to model construction were used as species-specific model tuning is difficult in multiple species modeling [21,55,64]. Models were run individually for 14 IAPS and the average fitted models were projected to the future climate scenarios. The niche distribution of each individual species was examined in ArcMap (version 10.4.1) for further analysis.

### 2.6. Evaluating Model Performance

The overall model performance was determined based on two statistics, the threshold-independent area under the receiver operating characteristic curve (AUC) [81,82] and the threshold-dependent true skill statistic (TSS) [83]. Model performance was considered as ‘good’ only if both measures were satisfied. The suitable area for individual species was determined by a binary threshold value which discriminates presences (suitable) and absences (not suitable) in grid cells. An ideal threshold determining approach should comply with bigger sensitivity and specificity values meaning smaller false negatives rate and false positives rate in turn in the evaluation data set [84]. Maximizing the sum of sensitivity and specificity approach which satisfies the 3 main threshold selection criteria (objectivity, equality and discriminability) has been recommended for models that use presence-only data and background or pseudo-absence data (i.e., MaxEnt) [85,86]. This threshold has been previously used in several modeling studies to discriminate the suitable areas of invasive species [49,87]. The relative importance of predictor variables to the individual IAPS models was analyzed using variable contribution table and jackknife test [50,51,88].

### 2.7. Development of Climatic Suitability Maps 

Thus, the maximum training sensitivity plus specificity logistic threshold of MaxEnt was used with the average model output to develop binary presence-absence maps for each species. The ‘reclassify’ tool of the ArcMap was used to develop presence-absence maps with suitable areas above the threshold and not suitable areas below. The suitable area prediction of each species was individually examined and assessed with the ecology of the species for verification. The range size of each species was calculated using the field calculator by multiplying the number of cells of presence by the cell size. All classified 14 layers were summed up to develop a combined raster using the raster calculator tool of the spatial analyst toolbox. The combined raster layer (climatic ‘heat map’ combining climatic suitability of 14 IAPS) was further classified into five classes in ArcMap (manual classification). The maximum number of layers that overlapped at any one place was 8; thus, we set five classes as: very low (0 IAPS), low (1–2 IAPS), moderate (3–4 IAPS) high (5–6 IAPS) and very high (7–8 IAPS). The very high class consisted of the highest (7–8) number of IAPS overlapped while the very low category is predicted as free from IAPS. The same exercise was carried out with the average model result of individual species for future climate, RCP 4.5 and RCP 8.5 for 2050 and 2070. The five classes used for classifying the combined raster of current climate were used for classifying the combined rasters in the future climate as well. Therefore, we developed 5 maps combining climatic suitability of 14 IAPS under current and future climate. 

By 2050 and 2070, the areas of suitability changes were calculated for 5 classes and 14 species, under both RCP scenarios. The potential suitability of each class was further analyzed under three categories to identify the areas of contraction (area loss), expansion (area gain) and unchanged (the area not changed) and those calculations were also done by 2050 and 2070 under both scenarios. Area of suitability changes of 14 individual species and 5 classified classes were calculated as a percentage of the proportion of area increase in relation to the original area of the species/class ((original area – new area/original area) * 100). Therefore, area reductions were expressed as negative values. Further, changes in the area of suitability in three area change categories (i.e., contraction, expansion and unchanged) were calculated as a percentage of the proportion of the area of each category in relation to the original area of the respective suitability class ((area of category/original area of the respective class) * 100).

## 3. Results

### 3.1. Species Distribution Models of 14 IAPS 

The predictive performances of evaluated IAPS were acceptable based on the quantitative assessments of model performance, AUC and TSS statistics (Supplementary material S2). The mean AUC values of the 14 IAPS ranged from 0.788 (lowest) to 0.995 (highest) with an average of 0.922 (±0.06). Eleven IAPS out of 14 had AUC values > 0.9. The average TSS values of the 14 IAPS ranged from 0.456 (lowest) to 0.896 (highest) with an average of 0.7046 (±0.04). Four IAPS had TSS >0.8. We analyzed the relative contribution of predictor variables to the individual IAPS models. Three precipitation variables (precipitation of driest month, annual precipitation and precipitation seasonality) and two temperature variables (minimum temperature of coldest month and mean temperature diurnal range) appeared to be highly contributing to climatic suitability (Supplementary material S3). Jackknife test also confirmed the importance of the above variables; additionally, it identified precipitation seasonality as an important predictor. 

### 3.2. Climatic Suitability of Individual IAPS 

**Figure 2:**
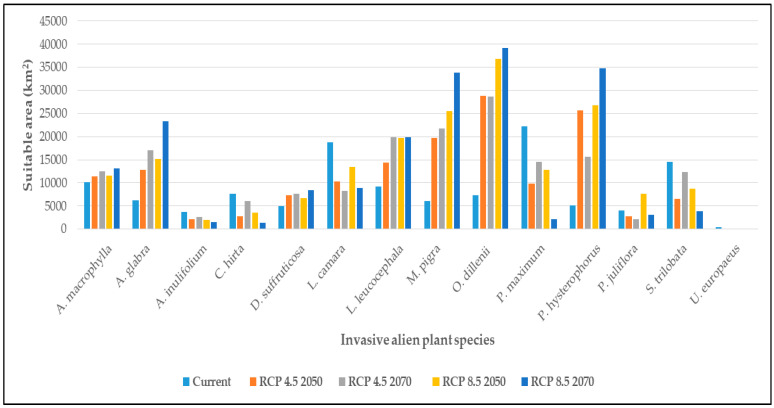
Projected area of suitability (km^2^) of the 14 priority IAPS in Sri Lanka under current climate and MIROC5 RCP 4.5 and RCP 8.5 for 2050 and 2070.

Under the current climate, both *P. maximum* and *L. camara* predicted the largest areas of 22,205 km^2^ and 18,851 km^2^ respectively. The species that was predicted to occupy the least area (471 km^2^) of suitability was *U. europaeus* (Figure 2, Supplementary material S4). Seven IAPS, namely *A. macrophylla*, *A. glabra*, *D. suffruticosa*, *L. leucocephala*, *M. pigra*, *O. dillenii* and *P. histerophorus* are projected to increase the overall climatic suitability in the future while the other seven IAPS, *A. inulifolium*, *C. hirta*, *L. camara*, *P. maximum*, *P. juliflora*, *S. trilobata* and *U. europaeus*, are projected to decrease. By 2050, all evaluated species are projected to change their respective suitability areas by more than 10% compared to the current climate under both scenarios, irrespective of whether it was a range expansion or a contraction. Five IAPS, *A. glabra*, *L. leucocephala*, *M. pigra*, *O. dillenii* and *P. histerophorus*, are projected to expand the range significantly in the future under both RCP scenarios. This expansion can be up to several fold by 2070 under RCP 8.5 compared to the present; for instance, *P. histerophorus* (581%), *M. pigra* (465%) and *O. dillenii* (440%). By 2070 *L. camara* is projected to loss overall suitability by 56% and 52% under RCP 4.5 and RCP 8.5 respectively compared to the current climate. Similarly, *P. maximum* is also projected to contract suitability by 35% and 90% under the above RCPs. It should be noted that by 2050 *U. europaeus* is projected to decrease its overall suitability by 100% compared to the current climate under both emission scenarios. 

### 3.3. Climatic Suitability for Multiple Species Establishment

**Figure 3:**
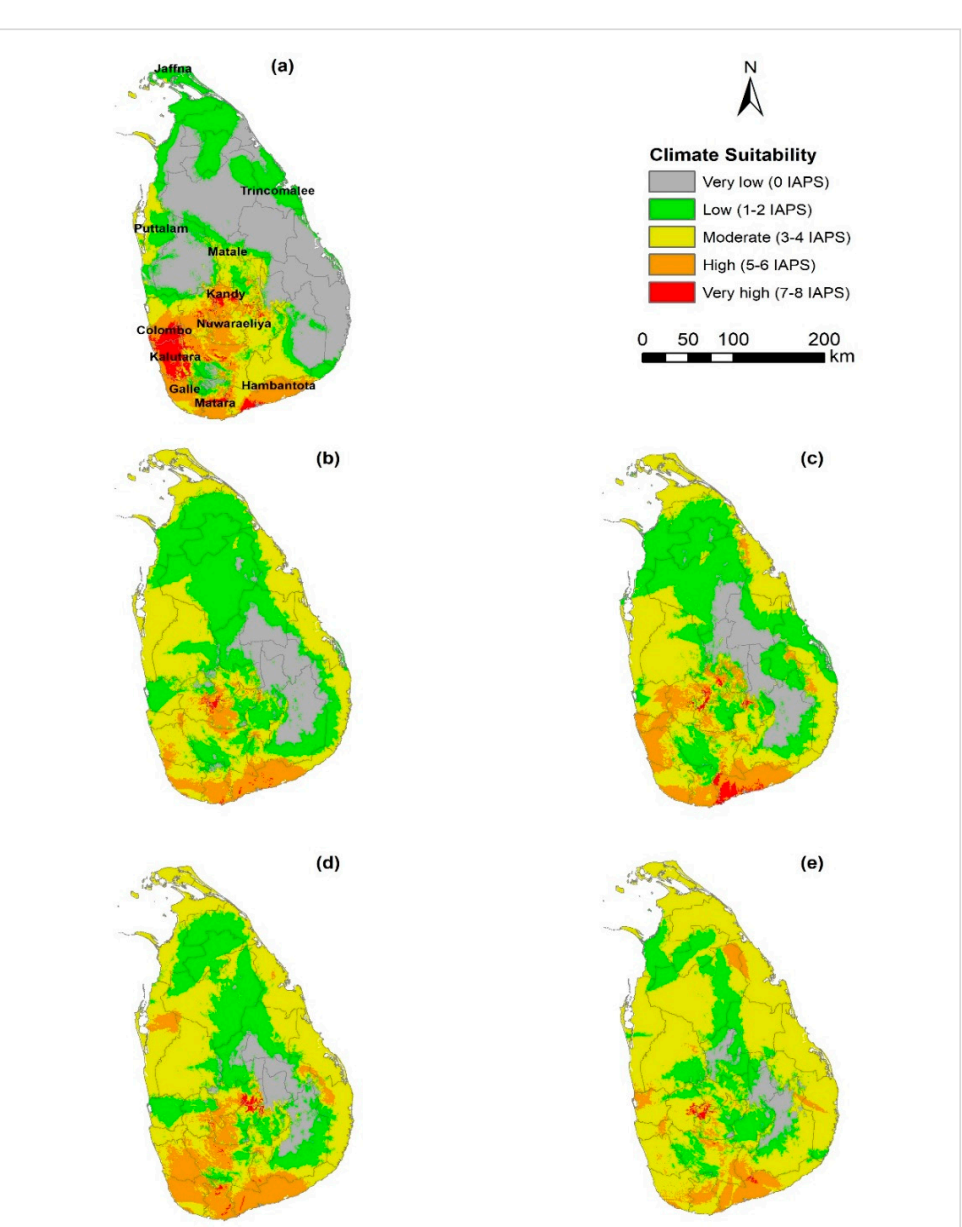
Maps showing current and future projected climatic suitability for 14 nationally important IAPS in Sri Lanka. (**a**) Current climate; (**b**) MIROC5 RCP 4.5 for 2050; (**c**) MIROC5 RCP 4.5 for 2070; (**d**) MIROC5 RCP 8.5 for 2050 and (**e**) MIROC5 RCP 8.5 for 2070. Areas climatically suitable for a relatively higher number of IAPS are denoted by hot colors (red) while the areas relatively less suitable by the cooler colors (green). Color codes used to signify climatic suitability classes are equivalent across five maps and denote same numbers of predicted IAPS.

**Figure 4:**
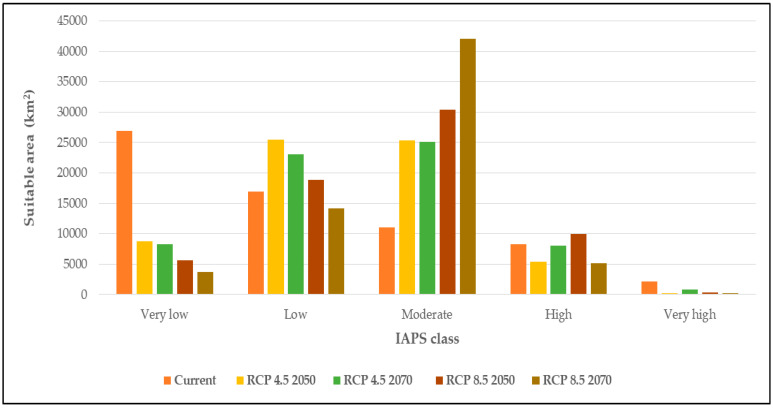
Projected area of suitability (km^2^) of the 5 classes of IAPS in Sri Lanka under current climate and MIROC5 RCP 4.5 and RCP 8.5 for 2050 and 2070. IAPS class = Invasive alien plant species class.

Figure 3 shows the combined map of climatic suitability (heat map) of 14 IAPS under current climate and RCP 4.5 and RCP 8.5 for 2050 and 2070. The projected map under current climate shows a region of potential IAPS concentration in the south and west parts of Sri Lanka (particularly in Colombo and Kalutara districts). In the future, this area is projected to become slightly less suitable for diverse IAPS. Under RCP 4.5, the very high suitability class (7–8 IAPS) is projected to have an overall decrease in the climatic suitability by 2050 and then increase by 2070, resulting in a 63% overall contraction of the suitable area relevant to current climate; while under RCP 8.5 the suitable area is projected to decrease continuously resulting in a 90% overall contraction (Figure 4, Supplementary material S5). Under RCP 4.5, the high suitability class (5–6 IAPS) is projected to decrease by 2050 and then increase by 2070 resulting in an overall 3% contraction of the suitable area compared to the current climate; while under RCP 8.5, this suitable area is projected to increase by 2050 and then decrease by 2070 resulting in an overall 38% contraction. The moderate suitability class (3–4 IAPS) is projected to increase substantially by 127% under RCP 4.5 and by 280% under RCP 8.5 by 2070 relative to the current climate. The projected area expansions in this class are significantly high by 2050 under both scenarios. The low suitability class (1–2 IAPS) is projected to increase by 2050 compared to the current climate and then decrease by 2070 under both RCPs, resulting in an overall 37% area expansion and 16% area contraction by 2070 compared to current climate under RCPs 4.5 and RCP 8.5 respectively. Generally, the very low suitability class (area free from IAPS) is projected to contract significantly by 2070, resulting in 69% and 86% overall contraction compared to the current climate under RCP 4.5 and 8.5 respectively. 

**Figure 5:**
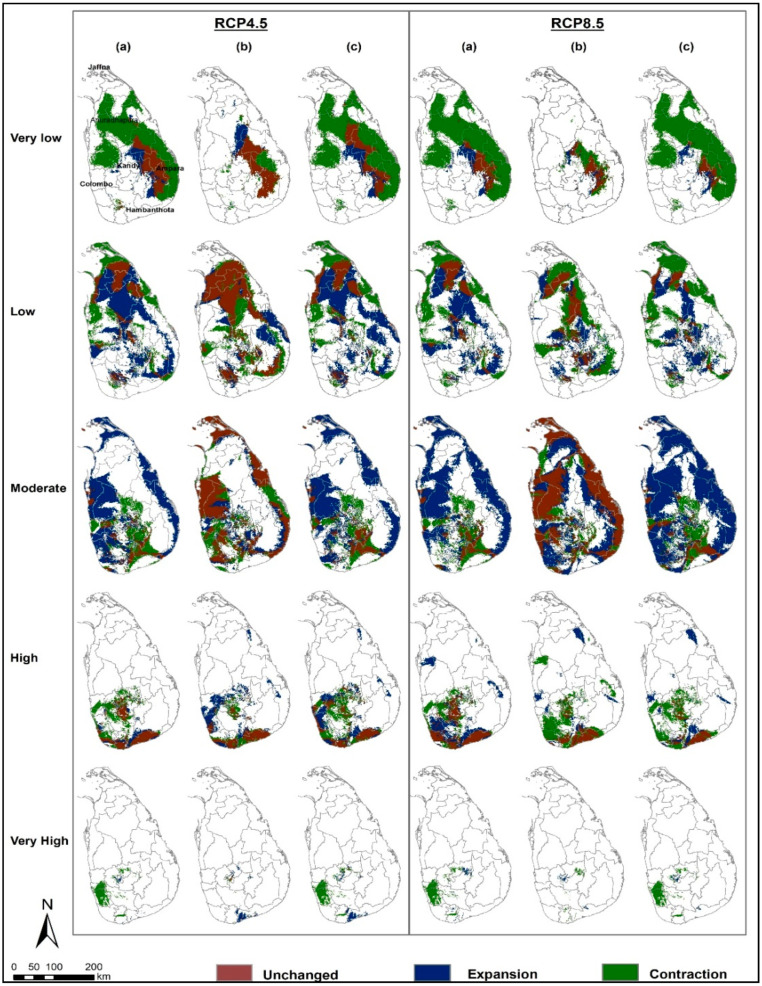
Projected potential distribution maps showing areas of contraction, expansion and the area unchanged (stable) of the 5 IAPS classes under RCP 4.5 and RCP 8.5 for 2050 and 2070 in Sri Lanka. (**a**) = 2050 compared to current climate; (**b**) = 2070 compared to 2050; (**c**) = 2070 compared to current climate.

Figure 5 and Table 3 illustrate the projected changes of the five IAPS suitability classes in terms of relative range expansion (gain), contraction (loss) and unchanged (stable) under climate change. In the very high suitability class, the projected range contractions are prominent (100%) under both RCP scenarios by 2050 compared to the current climate. These area contractions are predicted in the western part of the country in Colombo and Kalutara districts. However, under RCP 4.5, the very high suitability class shows a high gain by 2070 compared to 2050 (358%). Unchanged areas in the very high suitability class are predicted as substantially small. Also, the losses and gains in the high suitability class are predicted as significant and scattered in the central highlands, western to southern parts of the country; though a substantial area in the southern coastal lowlands shows unchanged suitability. The moderate class clearly displays more area gain than area loss which is prominent by 2050 than later. This class is projected to gain significant area in the coastal lowlands of the country while losing relatively a small area in the central parts and more downward to the south. In the low and moderate suitability classes, both area gains and losses are substantially high (>50%) by 2050 under both RCP scenarios. Very low class is predicted to contract in the future under both RCP scenarios. It should be noted that the area losses in this class were high compared to area gains and unchanged under both scenarios. 

## 4. Discussion

### 4.1. Species Distribution Models of 14 IAPS 

Based on AUC and TSS values, all evaluated species were considered for the analysis. AUC and TSS measures are generally used for evaluating SDMs in conservation planning [81,83]. The AUC ranges from 0 to 1 whereas TSS ranges from −1 to +1. In both cases, the upper levels imply perfect discrimination [51,83,89,90]. Generally, AUC values of <0.7 indicates poor performance; 0.7–0.9 moderate performance; and >0.9 high performance [89,91,92]. The TSS values <0.4 are considered as poor, 0.4–0.8 moderate and >0.8 very good model performance [93]. Threshold-dependent measures (i.e., TSS) are considered as more appropriate for defining suitable areas for invasive plants as it is based on the discrimination of presences and absences [83]. We did not calculate Cohen’s kappa due to its high dependency on prevalence (please read a study by [83] for details). We used spatial filtering to remove the sampling bias of occurrences. The effect of sampling bias can be addressed by constraining the background data to have the same bias as the sample data [90]. However, the bias file we prepared using the districts where the occurrences are distributed resulted in ecologically unrealistic model predictions for some species, far away from the training region. Therefore, the knowledge of species biology and distribution is crucial while selecting the most realistic model projection [57]. Moreover, a study by [94] recommend spatial filtering rather than background manipulation to correct sampling bias. The analysis of variable importance suggested that predicted habitat suitability was controlled by few temperature and precipitation variables in many IAPS species. Overall, precipitation variables showed a dominant control over the niche distribution of many IAPS as indicated by the contribution table (precipitation of driest month) and jackknife test (precipitation seasonality). Three variables contributed almost 90% of the model building in 10 out of the 14 IAPS evaluated. Changes in the suitability of IAPS could be mainly influenced by climatic factors that the MaxEnt model used and their compounding effects [49]. Therefore, we believe that the dependency of these IAPS on a few environmental variables (narrow range) may have brought about severe consequences on the potential distribution of these species under projected climate change.

### 4.2. Climatic Suitability of Individual IAPS 

In general, out of the 14 IAPS examined, seven species are projected to have increased climate suitability while the other seven species are projected to have decreased suitability. The projected climate suitability and the predicted changes are not uniform among the IAPS. A wide variation could be expected in terms of the rate of area changes and the pattern of potential suitability shift of the individual species (Supplementary material S4). However, in terms of overall area change, range expansions of the evaluated IAPS were significant compared to range contractions. Suitable area of *O. dillenii* is predicted to increase up to 4-fold by 2050 under both emission scenarios compared to the current climate. In other words, approximately half of the country’s total area (mainly dry lowlands) is predicted as suitable for *O. dillenii* invasion in the future. *P. histerophorus* is also predicted to have increased suitability (approximately 40% of the total area of the country) by 2050 in the north and east where many agricultural lands are located. The statistics under both scenarios indicates that *U. europaeus* could disappear from Sri Lanka by 2050.

In literature, many IAPS are projected to have increased potential area of suitability under changing climate [95]. Climate change can increase species invasion primarily by two processes (i) shifting propagules of invasive plants to a new location, (ii) deteriorating the resistance that native plants naturally possess against the establishment of invasive plants [96]. However, many distribution models have also predicted decreased suitability of invasive plants under projected climate change [95,97,98,99]. Literature shows the increased potential distribution of invasive plants in some countries in the future and decreasing trend of the same species in some other countries under projected climate change [100,101]. For instance, a study by [102] used SDMs to model the potential climate suitability of six aggressive IAPS (*Ageratum houstonianum*, *Chromolaena odorata*, *Hyptis suaveolens*, *Lantana camara*, *Mikania micrantha* and *Parthenium hysterophorus* across Nepal and found that these species will expand in the future. Likewise, a study by [49] evaluated the ecological niches of five troublesome IAPS in the Himalyan foothills to examine the invasive potential of these species. This study revealed that some invasive plants (*Ageratum conyzoides* and *Parthenium hysterophorus*) will show more conservative behavior in the novel climate while some other species (*Ageratina adenophora*, *Chromolaena odorata* and *Lantana camara*) will increase their spread. Decreased potential climate suitability for some IAPS implies that the relative ability to tolerate or cope with the climate change is low in those species; though invasive species show adaptations to thrive harsh environmental conditions [103]. Again, species invasion involves associations not only between invading species and the environment but also with other associated species too [104]. Therefore, this is considered as a complex phenomenon of several facets. Under the given high ecological and environmental tolerance, why some invasive species reduce their overall climate suitability in the future is not fully understood and adequately studied [49]. It could be partly explained by species-level invasive characteristics that are not shared among all invaders alike and strictly depends on the relationship between conditions of the environment and the individual species [104]. This means that the potential responses of invasive plants to climate change is different, inconsistent and needs to be studied to generate the most critical information for the strategic management of IAPS. 

### 4.3. Climate Suitability for Multiple Species Establishment

Western parts of Sri Lanka (particularly Colombo and Kalutara districts) are predicted to have very high suitability (7–8 IAPS) under current climate (Figure 3) which was relatively small and around 3% of the total land area of the country. According to climate projections, the suitability of this class is predicted to decrease in the future. This area loss could be mainly attributed by being converted into the moderate climate suitability by reduced suitability for one or two species, affecting the number of IAPS supported in the area. Similarly, the high climate suitability (5–6 IAPS) which accounts for approximately 13% of the total area of the country is predicted to decline and shift southward in the future. The moderate class (3–4 IAPS) is predicted to have significantly increased suitability under climate change. This area gain is predicted as an outward range expansion towards the coastal lowlands under both climate scenarios, but this expansion is severe under RCP 8.5. This increased suitability could be mainly attributed to the conversion of the very low and low suitability classes to the moderate class. This means more invasive species will have found this area suitable for establishment and spread in the future which is a concern in terms of invasiveness of the species considered. 

The low climate suitability class (1–2 IAPS) is predicted to shift inward under both scenarios in the future. This may be mainly by some expanding ranges of some IAPS or by new species colonization. The very low climate suitability (area free from IAPS invasion) is mainly distributed in the North-central and Eastern parts of the country under current climate (Figure 3a) and accounts for 41% of the total area. This area is predicted to decrease substantially and shift inwards (Figure 3b–e), suggesting the future invasion of one or several IAPS in this area. 

Overall, the total area represented by moderate, high and very high classes (>3 IAPS), which is about 33% of the area of the country, is predicted to increase by 45% and 58% under RCP 4.5 for 2050 and 2070 respectively; under RCP 8.5, these figures are as high as 90% and 121% respectively. Similarly, the total area represented by very low and low classes (<2 IAPS) which is about 67% of the area of the country, is predicted to decrease by 22% and 28% under RCP 4.5 and 44% and 59% under RCP 8.5 for 2050 and 2070 respectively. Both these total area changes, high suitability increase and low suitability decrease, signify the potentially increased risk of IAPS invasion in Sri Lanka in the future under projected climate change. The projected maps forecast a region of potential IAPS concentration in the west and south Sri Lanka. Most of those areas coincide with the lowland wet zone of the country where precipitation is relatively high and high level of biodiversity is concentrated. Therefore, the anticipated impacts of the projected changes on native biodiversity could be considerable. Similarly, a study by [63] has also shown that the vulnerability of island ecosystems to species invasion as invasion hotspots coincide with important biodiversity hotspots (i.e., islands, forest reserves) in India. However, literature shows that hotspots of invasive and the vulnerable native species can vary at different spatial scales [105].

The analysis of area changes has revealed critical information relevant to the range dynamics of these IAPS. Figure 5 confirms that both suitability expansions and contractions will simultaneously occur in all five classes (details in Table 3); though, it is expressed as an overall area increase or decrease in Figure 4. Those area losses and gains in the five classes are significantly high (>10%) under both RCP scenarios, suggesting a high rate of invasion dynamics under the projected climate change. Therefore, our results suggest that the overall risk of plant invasion attributed by these 14 IAPS could be intensified in Sri Lanka in the future. However, the distribution we observe in the future may be smaller than the forecast distribution as species dispersal is restricted by many other factors i.e., geographical barriers, human influences. 

Potential threats from IAPS that the oceanic islands will face in the future differ markedly with climate change [23] and thus, strategic interventions are needed to face the emerging challenges. It is not economically feasible to conserve the whole landscape from the detrimental effects of IAPS [106]. Thus, it is vital to know in which areas large numbers of IAPS can inhabit for directing limited resources [55,71]. Therefore, defining areas suitable for multiple IAPS establishment based on climate-based niche modeling can be considered as an increasingly important risk area prioritization approach in conservation planning. The climate suitability maps are powerful tools for policy makers and land managers to design suitable cost-effective strategies to control and manage IAPS. Projected maps clearly show exact locations across the landscape where urgent attention should be focused on. Such information is crucial to implement effective and successful control and management efforts. These results provide an invaluable insight into the climate suitability for multiple IAPS establishment in Sri Lanka.

### 4.4. Study Limitations

We do understand the limitations of our model predictions of 14 IAPS that were used for defining climate suitability for multiple IAPS establishment. SDMs are basically underpinned on several assumptions, ecological concepts and theories [16,78] and therefore uncertainty is a common issue associated with modeling techniques [17]. In distribution studies, the selection of GCMs could influence the projections of species distributions under climate change as different GCMs simulate environmental variables differently [17]. Performance of GCMs is difficult to evaluate and it may depend on variables used and the study region; thus, the use of multiple GCMs is often recommended in distribution modeling [107]. However, MIROC5 has been tested by previous empirical studies [66,108,109,110] for South Asia and also widely used in recent modeling literature in that region [49,111,112]. Single model uncertainty is also considered as an issue in predictive modeling. Studies have demonstrated that the variation in predictive performance among modeling techniques is significantly high and though the best model is applied it contributes uncertainty rather than other contributory factors, i.e., GCMs and RCPs [113]. Therefore, the use of a single model is increasingly criticized in SDM literature. Such uncertainty can be minimized through testing model performance across several modeling techniques and averaging them [114]. Therefore, multi-model approaches (i.e., Ensemble forecasting) are recommended for robust model predictions and also for cautious interpretation [115]. However, we used the well-established and widely recognized MaxEnt modeling technique as *it* frequently outperforms other presence-*only* modeling algorithms [46,55,116]. Further, this modeling study was undertaken entirely on bioclimatic variables based on the fact that climate controls the geographical distribution of plants predominantly over the other non-climatic factors [26] and we did not use non-climatic variables (i.e., disturbance, biotic interactions) to test our models. However, according to a study by [117], non-climatic variables of ecophysiological importance also drive the distribution of plants and failure to consider the contribution of all potentially important variables in models can affect the model accuracy and predictive ability. Therefore, we acknowledge the possible limitations generated by the exclusion of non-climatic predictors. Imperfect detection is also a common issue in presence-background (i.e., MaxEnt) models which is generally difficult to overcome in modeling studies and thus overlooked [118]. Imperfect detection can have important implications in predictive modeling resulting in two fundamental-error types in predictions: False-negative errors, (non-detection) and false-positive errors (misclassification) [119,120]. Therefore, it violates one of the fundamental assumptions of distribution modeling which is species are detected equally across the study site [121]. We used presence-only observed occurrences in our models which lack information on certain sites (i.e., unsampled, inaccessible, not yet occupied). Using MaxEnt as an example, a study by [118] has shown that imperfect detection influence on model discrimination of presence–background techniques. This issue may potentially lead to incorrect performance of distribution models and thus influence conservation decision making especially under potential climate change [16,122,123]. However, presence-only data (e.g., herbarium data) are commonly used when absence data are not available especially in less intensively sampled tropical countries [21]. Spatial predictions of modeling illustrate a species or habitat distribution in the geographical space rather than the ecological niche of species that exists in the environmental space [16]. Though interpreted by different names (i.e., habitat modeling, ecological niche modeling), all SDMs identify suitable habitat to support a species to survive and reproduce; however, this is not identical to ecological niche modeling [124].

## 5. Conclusions

The south and west parts of the country exhibited relatively high climate suitability for multiple IAPS establishment in the current climate and this region is predicted to remain in the future too. Similarly, the areas relatively free from IAPS, particularly north-central and south-eastern parts of Sri Lanka are predicted to increase suitability for IAPS. This implies that a vigilant surveillance system needs to be in place in these areas to obstruct pathways of introduction of new IAPS. Potential short-term and long-term impacts on native biodiversity and agriculture in these areas need to be realized for prior and timely actions. As in many modeling studies, our study also shows some limitations due to uncertainty generated by GCMs, SDMs and RCPs. Therefore, we endorse the importance of testing models through multiple SDMs, GCMs, and RCPs to better understand and quantify uncertainties caused by them and recognize the best approach to achieve a specific objective. We also recommend considering all potentially important predictors in model building and avoiding imperfect detection as far as possible, to improve the predictive performance of models. Possible implications that may arise due to these methodological deficits should be considered in decision-making. Therefore, additional effort is needed to minimize uncertainties for the reason that information is lacking or the underlying modeling processes are not fully understood. However, present results provide insight into the relative magnitude of the problem and an outline upon which surveillance, monitoring and control efforts should be focused on. Our findings specify areas of high climatic suitability for large numbers of IAPS and thus locations across the country where early detection and rapid response efforts need to be concentrated on. Therefore, we strongly recommend land managers to use this critical information to develop strategic control plans to combat these IAPS in Sri Lanka.

## Figures and Tables

**Table 1:** Details of 14 priority IAPS in Sri Lanka used for MaxEnt modeling. Adapted from [53].

No	Species	Family	Common Name	Life Form	Year of Introduction	Affected Climatic Zones/Habitats	No. of Occurrences
1	*Alstonia macrophylla* Wall.	Apocynaceae	Hard milkwood	Tree	unknown	Wet zone	116
2	*Annona glabra* L.	Annonaceae	Pond apple	Tree	unknown	Wet zone (e.g., Coastal wetlands)	69
3	*Austroeupatorium inulifolium* (H.B.K.) R. M. King & H. Rob	Asteraceae	Austroeupatorium	Shrub	unknown	Montane zone (e.g., Knuckles Conservation Forest)	60
4	*Clidemia hirta* (L.) D. Don	Melastomataceae	Soapbush, Koster’s curse	Herb	1894	Wet zone/Lowland wet zone forests (e.g., Sinharaja forest)	80
5	*Dillenia suffruticosa* (Griff ex Hook.f. & Thomson) Martelli	Dilleniaceae	Shrubby Dillenia	Tree	1882	Lowland wet zone	68
6	*Lantana camara* L.	Verbenaceae	Lantana	Bush	1826	Intermediate zone (e.g., Udawalawa National Park)	253
7	*Leucaena leucocephala* (Lam.) de Wit	Fabaceae	White lead tree	Shrub/Tree	1980	Dry and intermediate zones	151
8	*Mimosa pigra* L.	Fabaceae	Giant Mimosa	Bush	1980	Intermediate zone	36
9	*Opuntia dillenii* (Ker-Gawl.) Haw	Cactaceae	Prickly pear cactus	Cactus	unknown	Dry zone (e.g., Bundala National Park)	25
10	*Panicum maximum* Jacq.	Poaceae	Guinea grass	Grass	1801-1802	All zones	323
11	*Parthenium hysterophorus* L.	Asteraceae	Parthenium	Herb	1980	Dry zone	169
12	*Prosopis juliflora* (Sw.) DC.	Fabaceae	Mesquite	Tree	1880	Dry Zone (e.g., Bundala National Park)	48
13	*Sphagneticola trilobata* (L.) Pruski	Asteraceae	Creeping ox-eye	Herb	unknown	Wet zone	47
14	Ulex *europaeus* L.	Fabaceae	Gorse	Bush	1888	Montane zone/Wet Patana grassland (e.g., Horton Plains National Park)	15

**Table 2:** The selected subset of environmental variables used for MaxEnt modeling of 14 IAPS in Sri Lanka.

No	Variable	Abbreviation	Unit
1	Annual mean diurnal temperature range	bio2	°C
2	Maximum temperature of warmest month	bio5	°C
3	Minimum temperature of coldest month	bio6	°C
4	Annual precipitation	bio12	mm
5	Precipitation of driest month	bio14	mm
6	Precipitation seasonality	bio15	%
7	Precipitation of coldest quarter	bio19	mm

**Table 3:** Projected area of suitability (km^2^) of the 5 IAPS classes in terms of area contraction, expansion and unchanged under MIROC5 RCP 4.5 and RCP 8.5 for 2050 and 2070 (percentage changes are given within brackets).

IAPS Class	Suitable Area (km^2^) under RCP 4.5			Suitable Area (km^2^) under RCP 8.5		
	2050 (Relevant to Current Climate)	2070 (Relevant to Current Climate)	2070 (Relevant to 2050)	2050 (Relevant to Current Climate)	2070 (Relevant to Current Climate)	2070 (Relevant to 2050)
**Very Low**						
Contraction	21,181 (79)	21,700 (81)	2682 (31)	23,617 (88)	25,103 (93)	3055 (54)
Expansion	3019 (11)	3087 (11)	2231 (25)	2354 (9)	1847 (7)	1062 (19)
Unchanged	5738 (21)	5218 (19)	6074 (69)	3301 (12)	1816 (7)	2601 (46)
**Low**						
Contraction	9746 (58)	10,259 (61)	9170 (36)	11,677 (69)	13,177 (70)	11,943 (63)
Expansion	18,323 (109)	16,424 (97)	6757 (27)	13,693 (81)	10,482 (55)	7232 (38)
Unchanged	7134 (42)	6620 (39)	16,287 (64)	5203 (31)	3703 (20)	6953 (37)
**Moderate**						
Contraction	7247 (65)	6608 (60)	7649 (30)	7258 (66)	6443 (58)	5465 (18)
Expansion	21,574 (195)	20,624 (186)	7338 (29)	26,609 (240)	37,439 (338)	17,109 (56)
Unchanged	3823 (35)	4462 (40)	17,748 (70)	3811 (34)	4626 (42)	24,956 (82)
**High**						
Contraction	4429 (54)	4374 (53)	1992 (37)	3352 (41)	5988 (60)	7003 (71)
Expansion	1614 (20)	4151 (50)	4584 (84)	5015 (61)	2852 (29)	2205 (22)
Unchanged	3823 (46)	3878 (47)	3444 (63)	4899 (59)	2264 (23)	2911 (29)
**Very High**						
Contraction	2112 (100)	2065 (97)	105 (55)	2111 (100)	2109 (99)	354 (100)
Expansion	184 (9)	720 (34)	688 (358)	344 (16)	200 (9)	211 (60)
Unchanged	9 (0)	55 (3)	88 (46)	9 (0)	11(1)	0 (0)

## Supplementary Materials

The following are available online at https://www.mdpi.com/1099-4300/21/6/571/s1, S1: Results of Multicollinearity analysis of 19 bioclimatic variables. S2: Evaluation of model performances of 14 priority IAPS in Sri Lanka. S3: The three most highly contributed environmental variables to the MaxEnt model of 14 priority IAPS in Sri Lanka. S4: The potential distribution of Individual IAPS in the current climate and under MIROC5 RCPs 4.5 and 8.5 for 2050 and 2070. S5: Projected area of suitability (km^2^) of the 5 classes of IAPS in Sri Lanka under current climate and MIROC5 RCP 4.5 and RCP 8.5 for 2050 and 2070. Percentage changes are given relevant to current climate (percentage changes given within brackets are relevant to 2050).
