# Supplementary material for: Invasive Plant Species Establishment and Range Dynamics in Sri Lanka under Climate Change
Source: Entropy (Basel). 2019 Jun 5;21(6):571. doi: 10.3390/e21060571 (PMC7515060; doi:10.3390/e21060571)

**Electronic Supplementary Materials**

**Journal Name:** Entropy

**Title:** Climate suitability for multiple invasive alien plant species establishment in Sri Lanka under projected climate change and potential range dynamics

**Authors**: Champika S. Kariyawasam^1*^, Lalit Kumar^1^, Sujith S. Ratnayake^2^

^1^Ecosystem Management, School of Environmental and Rural Science, University of New England, Armidale, NSW 2351, Australia

^2^Climate Change Secretariat, Ministry of Mahaweli Development and Environment, Battaramulla 10120, Sri Lanka.

*Corresponding author: Champika S. Kariyawasam

Tel: +61-478686973; Fax: +61-267732769; Email: ckariyaw@myune.edu.au

**S1 Figure** Results of Multicolinearity analysis of 19 bioclimatic variables.

**
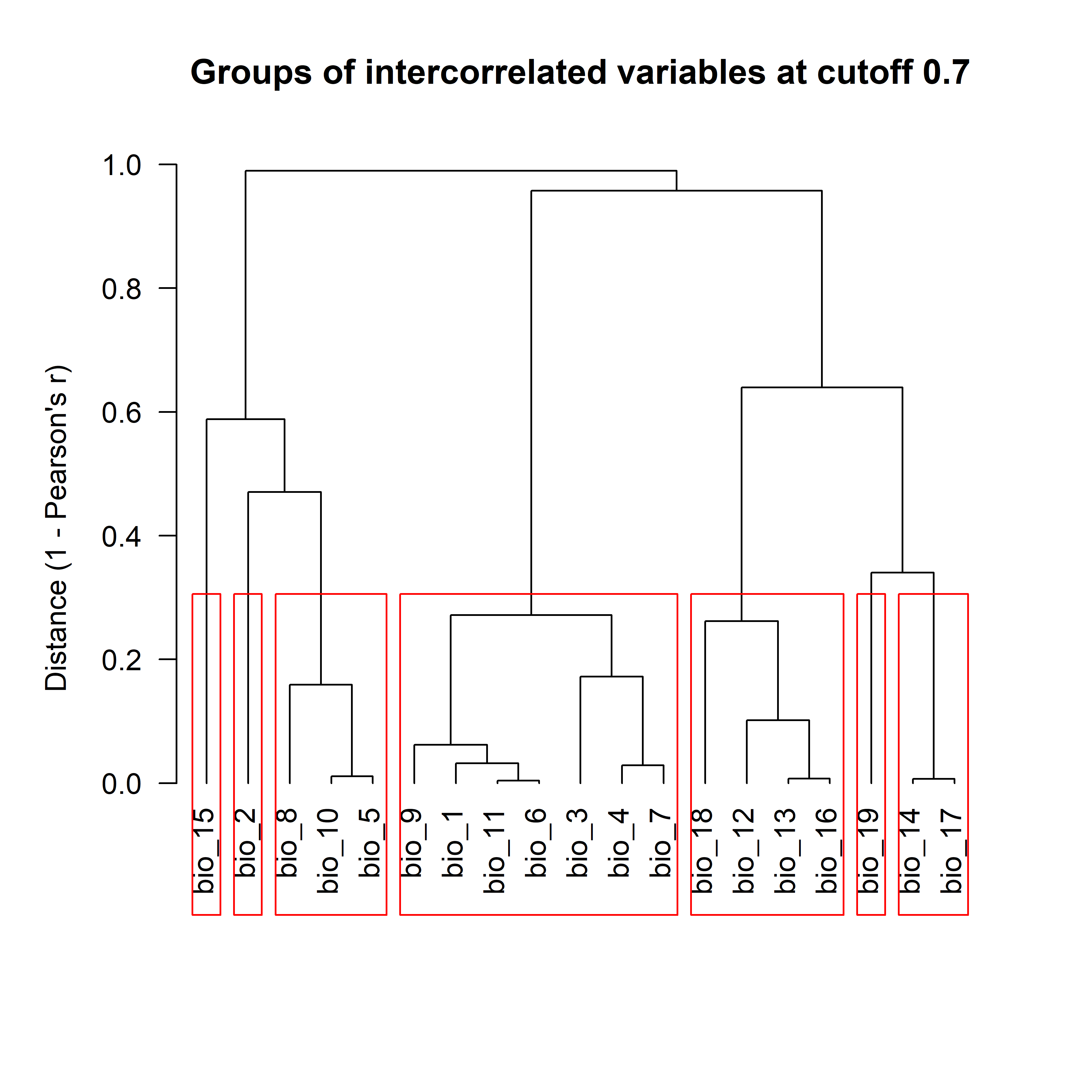
**

bio1 = Annual mean temperature; bio2 = Mean diurnal range (Mean of monthly (maximum temperature - minimum temperature)); bio3 = Isothermality (bio2/bio7) (* 100); bio4 = Temperature seasonality (standard deviation *100); bio5 = Maximum temperature of warmest month; bio6 = Minimum temperature of coldest month; bio7 = Temperature annual range (bio5-bio6); bio8 = Mean temperature of wettest quarter; bio9 = Mean temperature of driest quarter; bio10 = Mean temperature of warmest quarter; bio11 = Mean temperature of coldest quarter; bio12 = Annual precipitation; bio13 = Precipitation of wettest month; bio14 = Precipitation of driest month; bio15 = Precipitation seasonality (Coefficient of Variation); bio16 = Precipitation of wettest quarter; bio17 = Precipitation of driest quarter); bio18 = Precipitation of warmest quarter; bio19 = Precipitation of coldest quarter.

**S2 Table** Evaluation of model performances of 14 priority IAPS in Sri Lanka

|  | **Species** | **Mean AUC** | **Average TSS** | **Overall model performance** |
| --- | --- | --- | --- | --- |
| 1 | *Alstonia macrophylla* | 0.941 | 0.758 | Moderate |
| 2 | *Annona glabra* | 0.967 | 0.845 | Good |
| 3 | *Austroeupatorium inulifolium* | 0.984 | 0.896 | Good |
| 4 | *Clidemia hirta* | 0.948 | 0.739 | Moderate |
| 5 | *Dillenia suffruticosa* | 0.958 | 0.776 | Moderate |
| 6 | *Lantana camara* | 0.788 | 0.462 | Moderate |
| 7 | *Leucaena leucocephala* | 0.856 | 0.551 | Moderate |
| 8 | *Mimosa pigra* | 0.911 | 0.663 | Moderate |
| 9 | *Opuntia dillenii* | 0.934 | 0.618 | Moderate |
| 10 | *Panicum maximum* | 0.796 | 0.456 | Moderate |
| 11 | *Parthenium hysterophorus* | 0.946 | 0.820 | Good |
| 12 | *Prosopis juliflora* | 0.973 | 0.730 | Moderate |
| 13 | *Sphagneticola trilobata* | 0.910 | 0.659 | Moderate |
| 14 | *Ulex europaeus* | 0.995 | 0.892 | Good |

AUC is Area under the ROC (Receiver Operating Characteristic) Curve; TSS is True Skill Statistic. Overall model performance was evaluated considering both AUC and TSS values. Overall model performance was considered as ‘good’ if both AUC and TSS show good performance (AUC >0.9 and TSS > 0.8). Overall model performance was considered as ‘moderate’ if one measure shows moderate performance (AUC >0.7-0.9 or TSS 0.4-0.8) though the other measure performs well.

**S3 Table** The three most highly contributed environmental variables to the MaxEnt model of 14 priority IAPS in Sri Lanka.

| **No.** | **Species** | **Variable 1** | **Variable 2** | **Variable 3** | **Total contribution** |
| --- | --- | --- | --- | --- | --- |
| 1 | *Alstonia macrophylla* | bio14 (65.2%) | bio12 (25.6%) | bio6 (3.1%) | 93.9% |
| 2 | *Annona glabra* | bio14 (49.6%) | bio2 (26.1%) | bio12 (11.4%) | 87.1% |
| 3 | *Austroeupatorium inulifolium* | bio6 (73.9%) | bio5 (10.1%) | bio14 (6%) | 90% |
| 4 | *Clidemia hirta* | bio14 (77.4%) | bio12 (12.6%) | bio2 (3.4%) | 93.4% |
| 5 | *Dillenia suffruticosa* | bio12 (50.5%) | bio14 (42.6%) | bio6 (2.1%) | 95.2% |
| 6 | *Lantana camara* | bio5 (32.8%) | bio14 (20.5%) | bio15 (18.9%) | 72.2% |
| 7 | *Leucaena leucocephala* | bio5 (24.4%) | bio15 (21.6%) | bio14 (19.3%) | 65.3% |
| 8 | *Mimosa pigra* | bio2 (28.5%) | bio5 (27.8%) | bio12 (22.6%) | 78.9% |
| 9 | *Opuntia dillenii* | bio12 (69.2%) | bio6 (19.5%) | bio2 (8.2%) | 96.9% |
| 10 | *Panicum maximum* | bio15 (36%) | bio14 (18.8%) | bio19 (18%) | 72.8% |
| 11 | *Parthenium hysterophorus* | bio15 (86.5%) | bio2 (5.9%) | bio12 (2.6%) | 95% |
| 12 | *Prosopis juliflora* | bio12 (74.7%) | bio14 (13.1%) | bio_6 (4.9%) | 92.7% |
| 13 | *Sphagneticola trilobata* | bio14 (79.3%) | bio5 (7%) | bio15 (4.4%) | 90.7% |
| 14 | *Ulex europaeus* | bio6 (87.1%) | bio15 (10.3%) | bio12 (1.4%) | 98.8% |

**S4 Table** Projected area of suitability (km^2^) of the 14 priority IAPS in Sri Lanka under current climate and MIROC5 RCP 4.5 and RCP 8.5 for 2050 and 2070. Percentage changes are given relevant to current climate (percentage changes given within brackets are relevant to 2050).

| **Species** |  | |  | | | **Suitable area (km^2^)** | | |  | | |  | |
| --- | --- | --- | --- | --- | --- | --- | --- | --- | --- | --- | --- | --- | --- |
|  | **Current** | **RCP 4.5**  **2050** | | **% change** | **RCP 4.5 2070** | | **% change** | **RCP 8.5 2050** | | **% change** | **RCP 8.5 2070** | | **% change** |
| *A. macrophylla* | 10132 | 11383 | | 12 | 12512 | | 23 (10) | 11563 | | 14 | 13194 | | 30 (14) |
| *A. glabra* | 6253 | 12872 | | 106 | 17023 | | 172 (32) | 15147 | | 142 | 23392 | | 274 (54) |
| *A. inulifolium* | 3672 | 2089 | | -43 | 2630 | | -28 (26) | 1913 | | -48 | 1464 | | -60 (-23) |
| *C. hirta* | 7563 | 2712 | | -64 | 6029 | | -20 (122) | 3558 | | -53 | 1344 | | -82 (-62) |
| *D. suffruticosa* | 4931 | 7313 | | 48 | 7687 | | 56 (5) | 6661 | | 35 | 8408 | | 71 (26) |
| *L. camara* | 18851 | 10274 | | -45 | 8234 | | -56 (-20) | 13441 | | -29 | 8959 | | -52 (-33) |
| *L. leucocephala* | 9180 | 14396 | | 57 | 19926 | | 117 (38) | 19778 | | 115 | 19933 | | 117 (1) |
| *M. pigra* | 6000 | 19740 | | 229 | 21732 | | 262 (10) | 25586 | | 326 | 33920 | | 465 (33) |
| *O. dillenii* | 7268 | 28836 | | 297 | 28709 | | 295 (0) | 36797 | | 406 | 39221 | | 440 (7) |
| *P. maximum* | 22205 | 9831 | | -56 | 14511 | | -35 (48) | 12820 | | -42 | 2190 | | -90 (-83) |
| *P. hysterophorus* | 5116 | 25661 | | 402 | 15599 | | 205 (-39) | 26770 | | 423 | 34819 | | 581 (30) |
| *P. juliflora* | 4007 | 2787 | | -30 | 2070 | | -48 (-26) | 7699 | | 92 | 3120 | | -22 (-59) |
| *S. trilobata* | 14594 | 6475 | | -56 | 12420 | | -15 (92) | 8809 | | -40 | 3898 | | -73 (-56) |
| *U. europaeus* | 471 | 0 | | -100 | 0 | | -100 (0) | 0 | | -100 | 0 | | -100 (0) |

Area reductions are denoted by negative (-) sign

**S5 Table** Projected area of suitability (km^2^) of the 5 classes of IAPS in Sri Lanka under current climate and MIROC5 RCP 4.5 and RCP 8.5 for 2050 and 2070. Percentage changes are given relevant to current climate (percentage changes given within brackets are relevant to 2050).

| **IAPS class** | **Suitable area (km^2^)** | | | | | | | | |
| --- | --- | --- | --- | --- | --- | --- | --- | --- | --- |
|  | **Current** | **RCP 4.5 2050** | **% change** | **RCP 4.5 2070** | **% change** | **RCP 8.5 2050** | **% change** | **RCP 8.5 2070** | **% change** |
| **Very low** | 26918 | 8756 | -67 | 8305 | -69 (-5) | 5655 | -79 | 3663 | -86 (-35) |
| **Low** | 16879 | 25457 | 51 | 23044 | 37 (-9) | 18896 | 12 | 14185 | -16 (-25) |
| **Moderate** | 11070 | 25397 | 129 | 25086 | 127 (-1) | 30420 | 175 | 42065 | 280 (38) |
| **High** | 8251 | 5437 | -34 | 8029 | -3 (48) | 9914 | 20 | 5116 | -38 (-48) |
| **Very high** | 2120 | 192 | -91 | 776 | -63 (304) | 354 | -83 | 211 | -90 (-40) |

Area reductions are denoted by negative (-) sign

**S6 Figure** The potential distribution of Individual IAPS in the current climate and under MIROC5 RCPs 4.5 and 8.5 for 2050 and 2070.


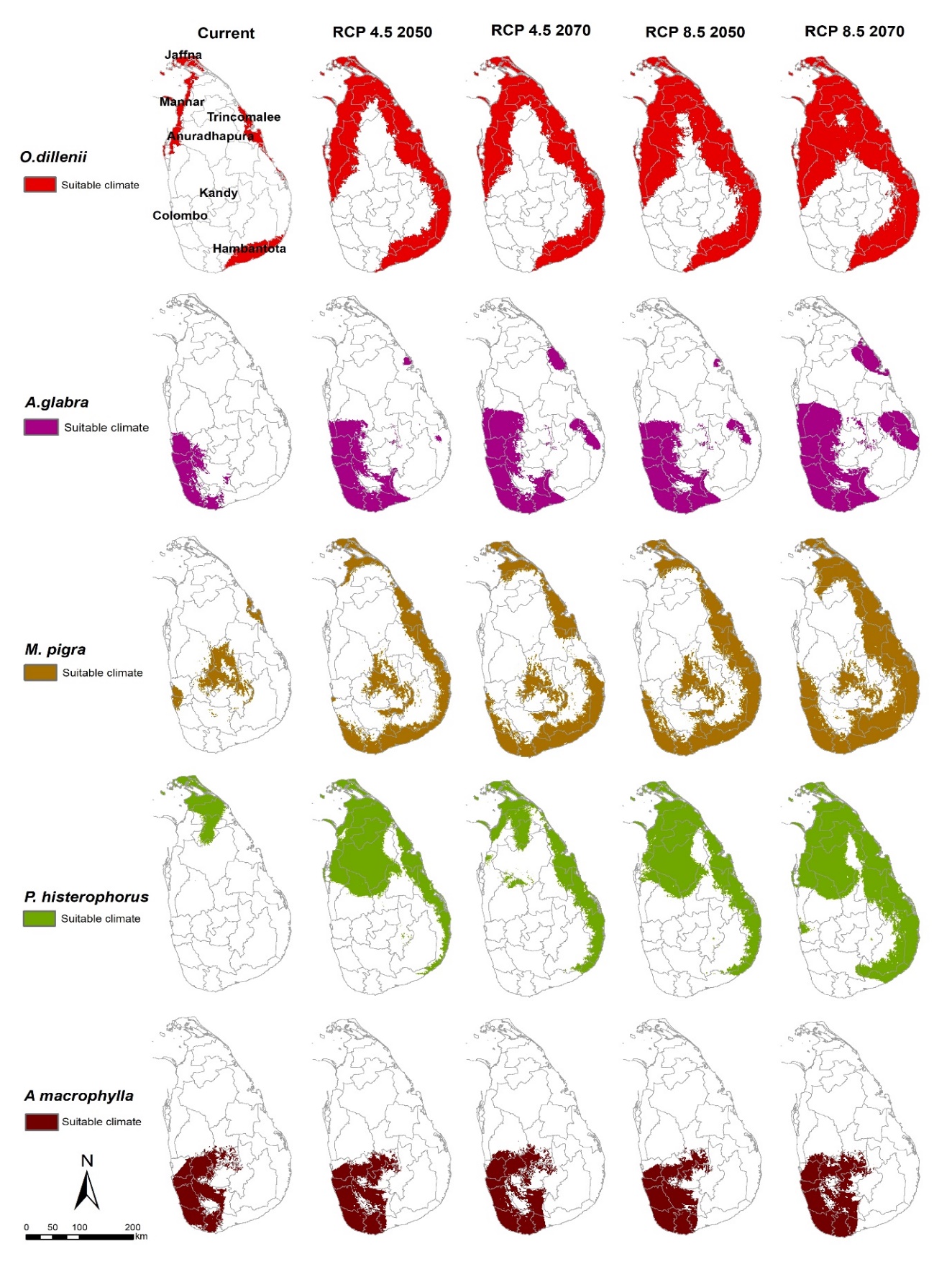


**S6 Figure** The potential distribution of Individual IAPS in the current climate and under MIROC5 RCPs 4.5 and 8.5 for 2050 and 2070 continued…


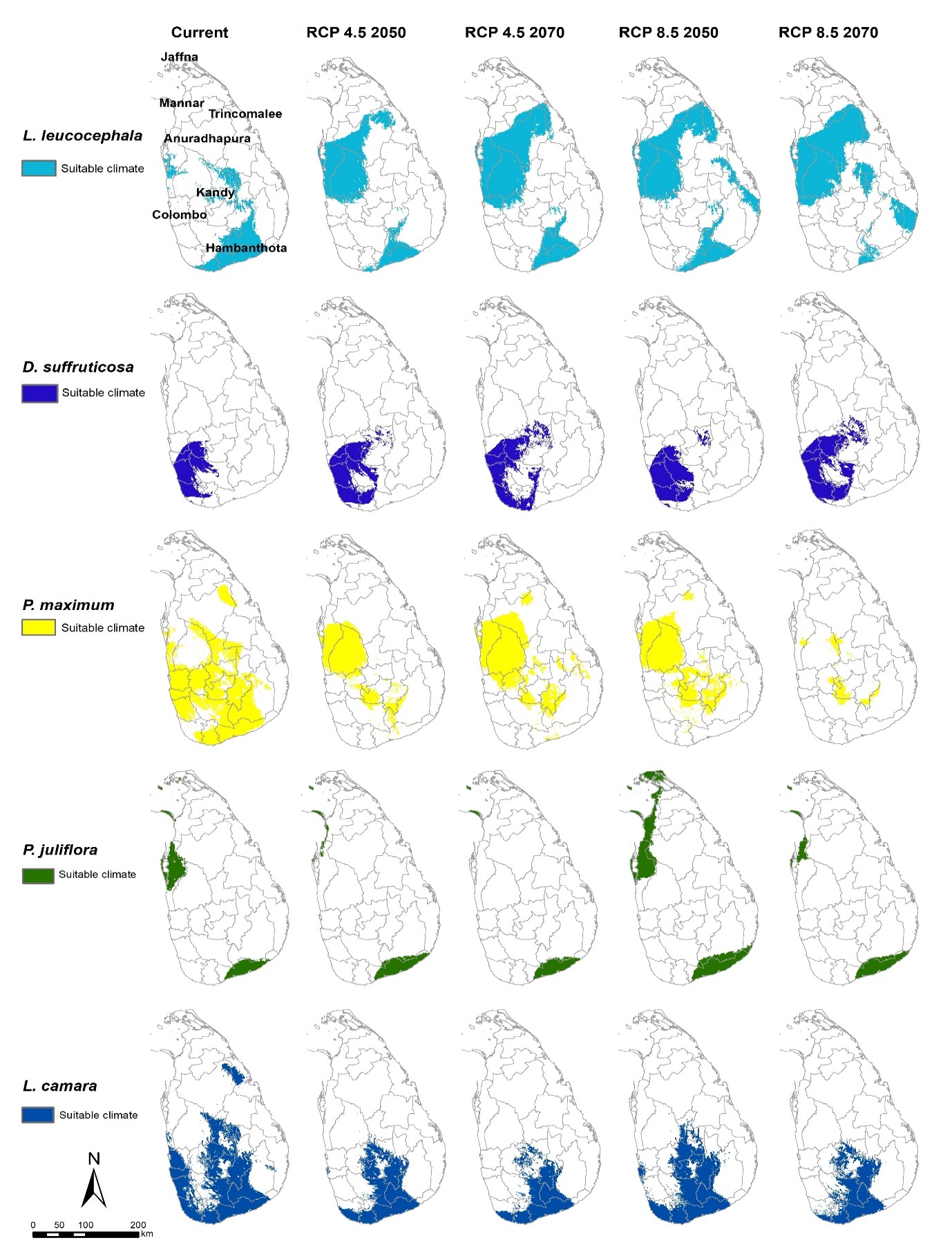


**S6 Figure** The potential distribution of Individual IAPS in the current climate and under MIROC5 RCPs 4.5 and 8.5 for 2050 and 2070 continued…


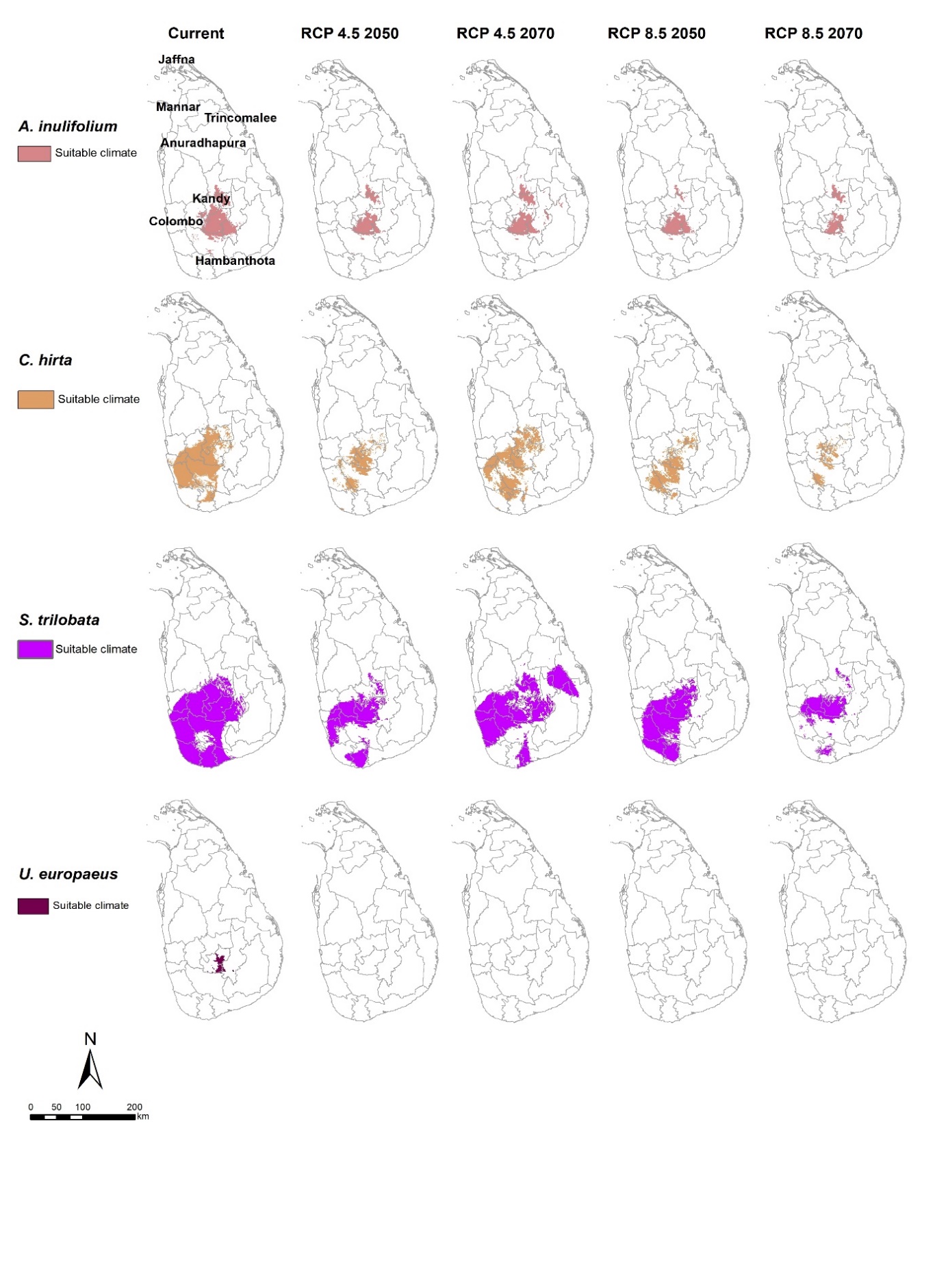

Supplement: Supplementary file 1 [file entropy-21-00571-s001.zip › Supplementary materials/Supplementary Materials.docx]
