# Supplementary figures and images for: Invasive Plant Species Establishment and Range Dynamics in Sri Lanka under Climate Change
Source: Entropy (Basel). 2019 Jun 5;21(6):571. doi: 10.3390/e21060571 (PMC7515060; doi:10.3390/e21060571)

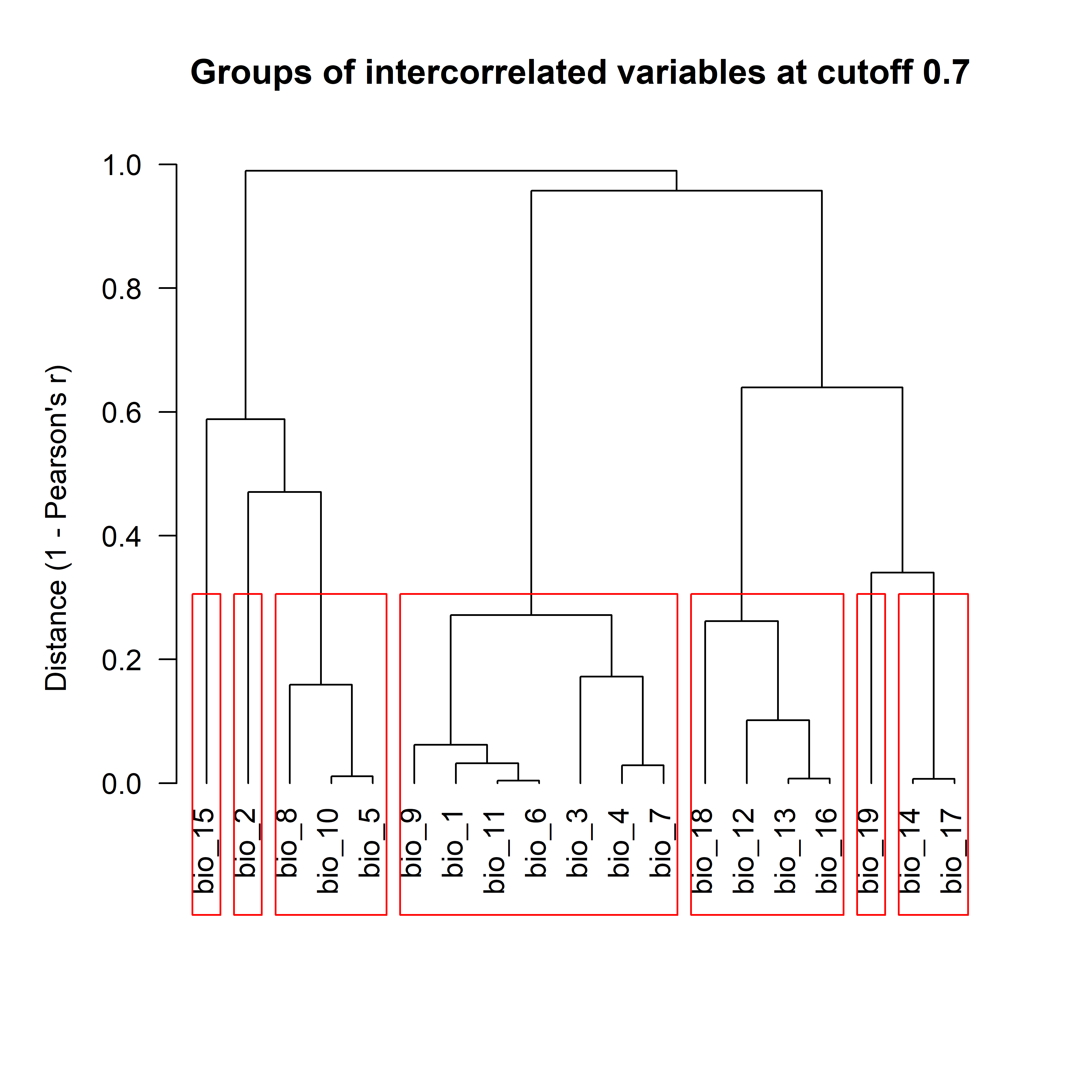

Supplement: Supplementary file 1 [file entropy-21-00571-s001.zip › Supplementary materials/S1 Figure.tiff]

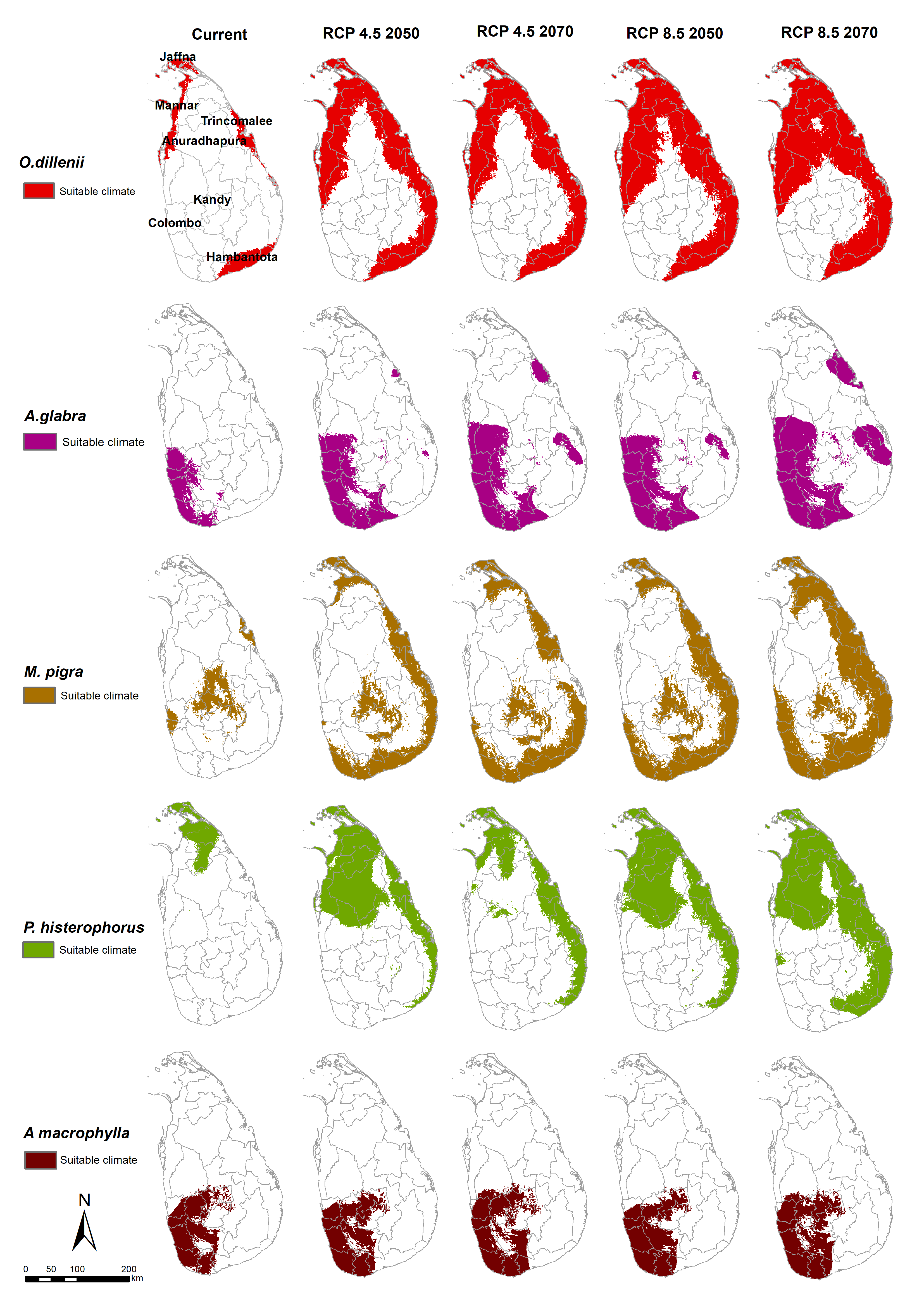

Supplement: Supplementary file 1 [file entropy-21-00571-s001.zip › Supplementary materials/S6 Figure_1.tif]

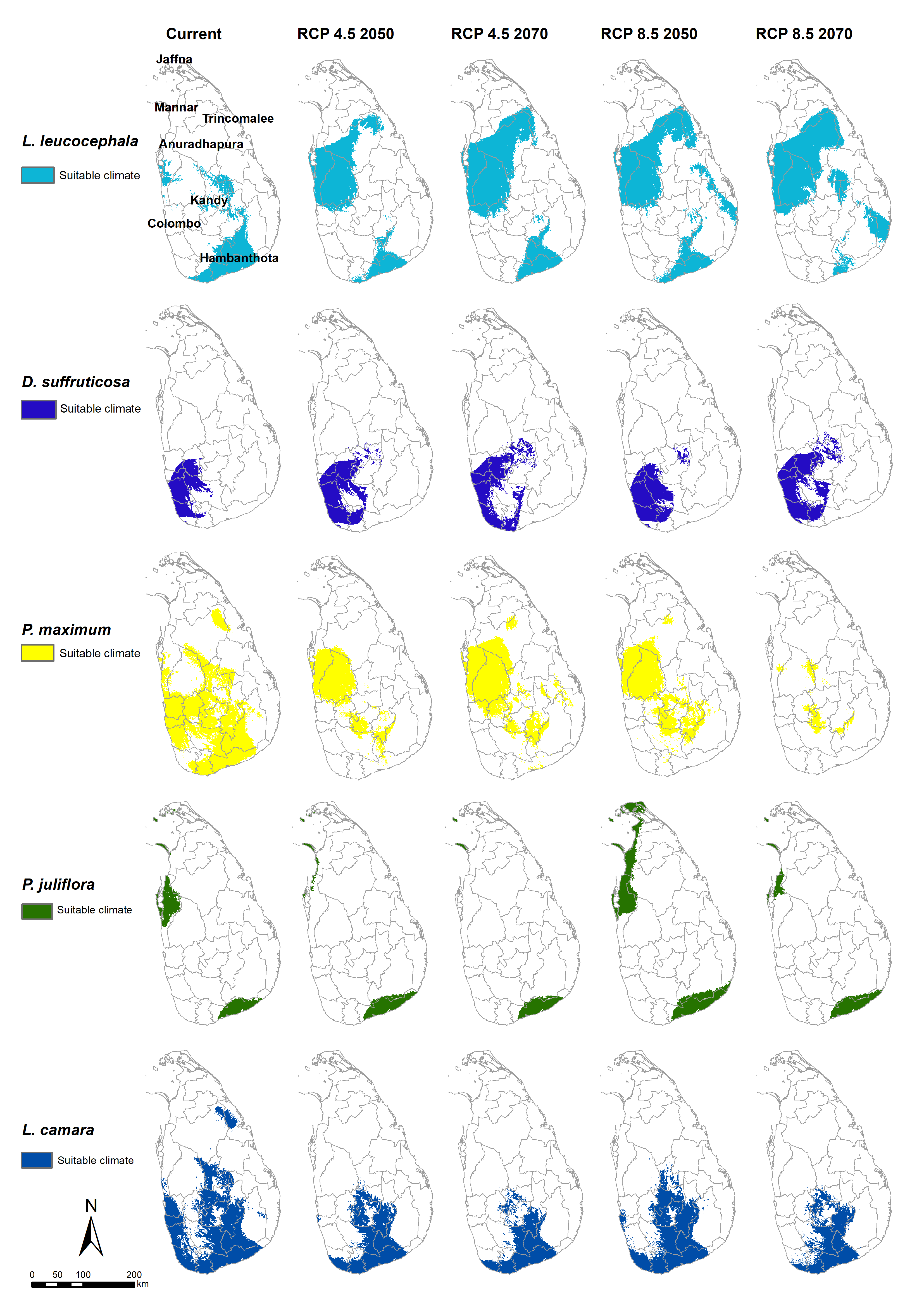

Supplement: Supplementary file 1 [file entropy-21-00571-s001.zip › Supplementary materials/S6 Figure_2.tif]

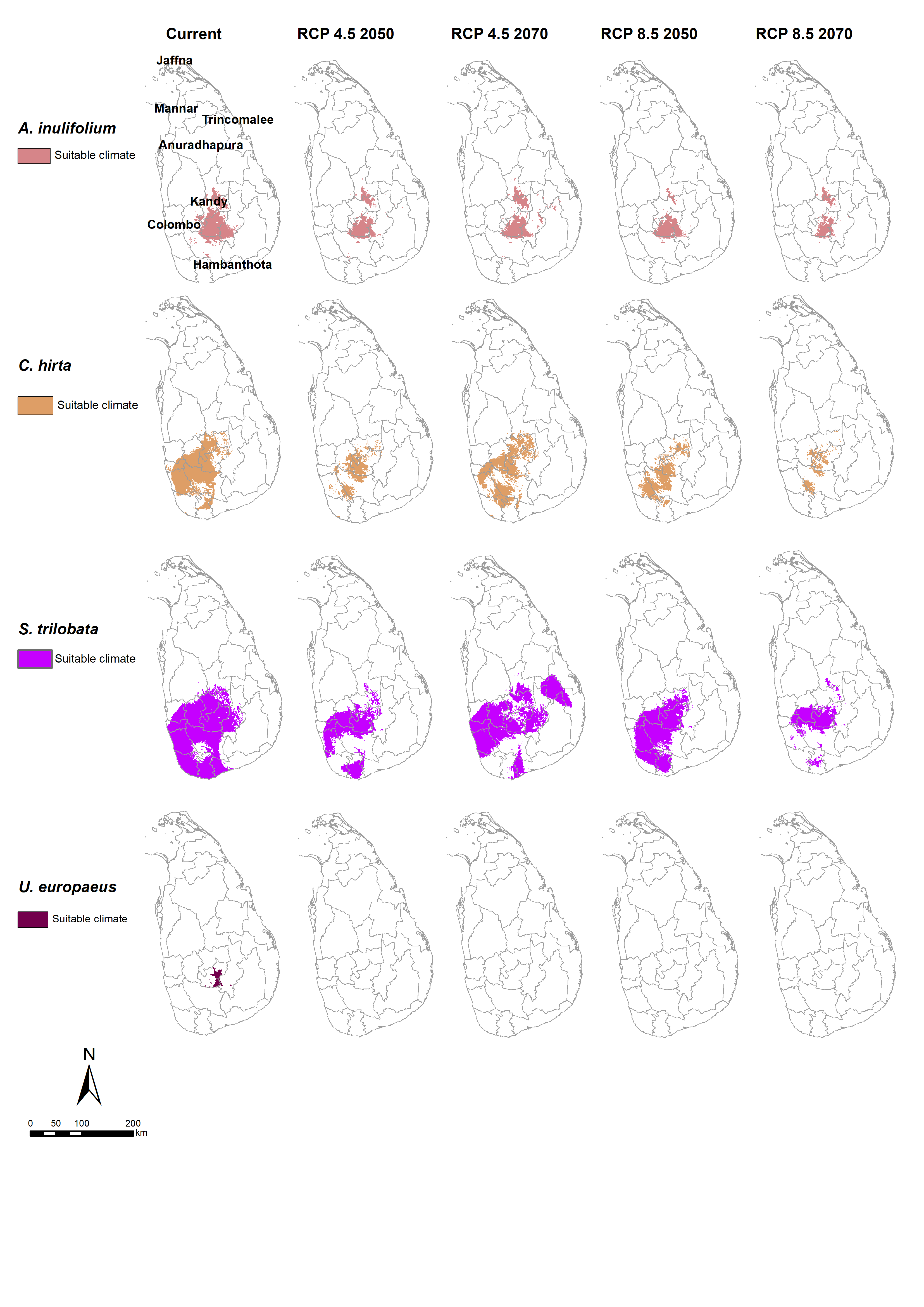

Supplement: Supplementary file 1 [file entropy-21-00571-s001.zip › Supplementary materials/S6 Figure_3.tif]
